# Dynamics of eye movements under time varying stimuli

**DOI:** 10.16910/jemr.11.1.6

**Published:** 2018-06-13

**Authors:** Verica Radisavljevic-Gajic

**Affiliations:** Villanova University, Villanova, USA

**Keywords:** Oculomotor model, ocular convergence, response dynamics, neural control

## Abstract

In this paper we study the pure-slow and pure-fast dynamics of the disparity convergence of the eye movements second-order linear dynamic mathematical model under time varying stimuli. Performing simulation of the isolated pure-slow and pure-fast dynamics, it has been observed that the pure-fast component corresponding to the eye angular velocity displays abrupt and very fast changes in a very broad range of values. The result obtained is specific for the considered second-order mathematical model that does not include any saturation elements nor time-delay elements. The importance of presented results is in their mathematical simplicity and exactness. More complex mathematical models can be built starting with the presented pure-slow and pure-fast first-order models by appropriately adding saturation and time-delay elements independently to the identified isolated pure-slow and pure-fast first-order models.

## Introduction

Studying dynamics of eye movements plays an important role in the development of various eye therapies (1) and provides useful information about understanding of neurological processes and the human brain function, (13, 18). Modeling of the disparity convergence has been studied in several papers (2, 4, 5, 6, 9, 10, 11, 12, 14). In some studies (3, 15, 17, 19) different problem formulations are used. Some of the papers have observed experimentally and analytically the presence of the slow and fast eye movement dynamics, (5, 11, 12, 14, 17, 19) . The analytical observation was made using the corresponding secondorder mathematical model (6, 9). 

This paper is a continuation of our previous paper (19), originally done for the constant eye stimuli using the second-order dynamic mathematical model derived in Alvarez et al. (6). For the model of Alvarez et al. (6), we perform exact mathematical analysis with the goal to isolate the slow and fast components, and present simulation results for the case of time varying eye stimuli since they produce some interesting phenomena not previously observed for the case of constant eye stimuli (19). The importance of presented results is in their mathematical simplicity and exactness. The results obtained and conclusions drawn are specific for the considered linear second-order mathematical model. By no means, in this study, we make an attempt to compete with more complex nonlinear models that might be of higher dimensions and include saturation and time-delay elements. Those models can produce results that more closely match the experimental results, but they have great difficulty in isolating slow and fast motions. In the study performed, isolation of slow and fast dynamic components is done analytically using exact (not approximative) mathematics.

## Disparity convergence eye movement and its slow and fast dynamics

In this section, we review the main results of Radisavljevic-Gajic (19) that will be used in this paper to study the disparity convergence eye movements under time varying eye stimuli. The linear dynamic mathematical model was derived for the disparity convergence eye dynamics in Alvarez et al. (6), page 384, formula (1), (see also Hung (10), page 252 for justification of the use of the second-order model)

**(1):**
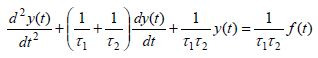


*y*(*t*) represents the eye position in degrees, *f *(*t*) is the eye stimules in degrees with respect to reference eye position, eye target position. The time constants in (1) are τ_1 _=224ms and τ_2 _=13 ms. They define respectively the slow τ_*s *_=τ_1_ and fast τ_*f *_=τ_2_ eye time constants (6), which motivated research of Radisavljevic-Gajic (19) to separate the coupled slow and fast dynamics into isolated pure-slow and pure-fast decoupled (independent) dynamics using theory of two-time scale dynamic systems (also known in differential equations and control engineering as theory of singular perturbations (16)). It is interesting to observe that the use of the second-order model is justified in Horng et al. (9), where a first-order model is used to represents the vergence oculomotor plant, see Figure 2 of that paper. In the follow-up of this paper (see Comment 1), we will show that the first-order model of Horng et al. (9) approximately represents the slow variable of the second-order model considered in this paper and defined in (1).

The second-order differential equation (1) is first converted into the state space form (8), by using the following change of variables *x*_1_(*t*)= *y*(*t*) and *x*_2_(*t*) =*dy*(*t*)/*dt *, producing

**(2):**
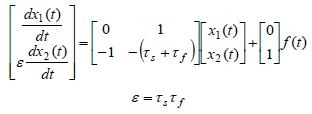


Since *x*_1_(*t*) represents the eye position, the variable *x*_2_(*t*) =*dx*_1_(*t*)/*dt* represents the eye angular velocity. The small parameter τ_*s*_τ_*f *_=ε that multiplies the first derivative of *x*_2_(*t*) is known as the singular perturbation parameter (16). Its very small value of ε= 0.002912 ≈ 0.003 indicates that the fast and slow dynamics are very well separated having the fast state variable *x*_2_(*t*) to be much faster than the slow state variable *x*_1_(*t*). However, due to coupling in (2), the slow variable *x*_1_(*t*) contains some portion of the fast variable and the other way around. Our goal is to exactly separate state variables and obtain pure-slow and pure-fast subsystems that are dynamically decoupled, from which we will be able to obtain information about the time evolution of pure-slow and pure-fast variables.

The slow and fast variables can be dynamically separated by using the very well-known Chang transformation (7), given by

**(3):**
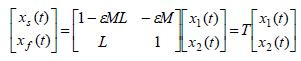


Constants *L *and *M* are obtained by solving the algebraic equations

**(4):**
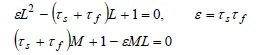


Applying (3) to (2) produces the decoupled pure-slow and pure-fast subsystems with a common input, that is

**(5):**
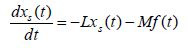


**(6):**
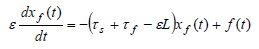


The quadratic algebraic equation for *L* has two solutions. It can be shown that the acceptable solution (19) is,

**(7):**
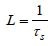


Having obtained the value for *L*, the solution for the M-equation is given by

**(8):**
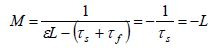


Using (7) and (8) in (5) and (6), produces two firstorder differential equations for pure-slow and pure-fast variables, whose coefficients are given in terms of the corresponding slow and fast time constants

**(9):**
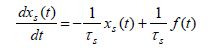


**(10):**
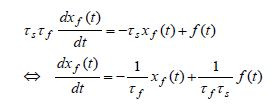


It should be observed that the pure-slow dynamics if determined only by the slow time constant, which is naturally expected. However, the pure-fast dynamics is determined by both the fast and slow time constants. Having obtained separated pure-slow and pure-fast mathematical models (9)-(10), the eye slow and fast dynamics can be independently studied and better understood since the coupling between slow and fast subsystems is eliminated.

The inverse Chang transformation relates the original state variables and the pure-slow and pure-fast variables obtained from (9)-(10) via the inverse Chang transformation (7), given by

**(11):**
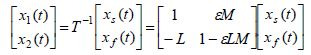


which leads to

**(12):**
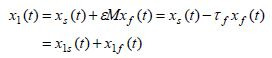


**(13):**
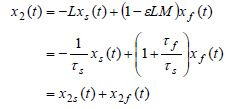


In the next section we perform simulation study of the pure-slow and pure-fast first-order models (9) and (10), and corresponding state variables (12) and (13) (eye position and eye angular velocity) given in terms of solutions of (9) and (10). In the future studies, one might consider using saturation and time-delay elements in either (9) and/or (10) to match better experimental results, and going backwards to the original coordinates (or using MATLAB/Simulink block diagrams) develop new nonlinear and higher dimensional mathematical models that have better agreements with experimental results.

## Pure-slow pure-fast subsystems under time varying stimuli

The constant input responses for the pure-slow and pure-fast dynamics with zero initial conditions) were considered in Radisavljevic-Gajic (19). In this section, we consider the eye stimuli force as a time varying function. We assume that the force changes periodically from 30 to 10 degrees every two seconds during the time interval of 10 seconds, that is

**(14):**
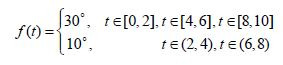


Using the given numerical data for the time constants the pure-slow and pure-fast mathematical models are given by

**(15):**
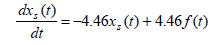


**(16):**
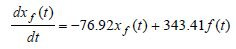


The transfer function (8), of the pure-slow (15) and pure-fast (16) subsystems are respectively given by

**(17):**
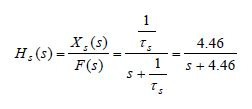


**(18):**
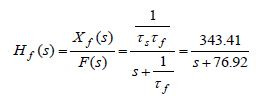


where *X *_*s *_(*s*), *X *_*f *_(*s*), *F*(*s*)are the Laplace transforms of the corresponding signals.

*Comment 1:* It is interesting to observe that in Horng et al. (9), a first-order model is used to represents the vergence oculomotor plant, with the transfer function defined by

**(18a):**
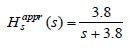


This approximate first-order model completely ignores the presence of the fast dynamics in the system. The approximate slow dynamics that partially includes information about the fast dynamics can be obtained from (2) by simply setting ε=0 in the second equation, which leads to the more accurate approximate slow subsystem than the one considered in Horng et al. (9), represented by one differential and one algebraic equation

**(18b):**
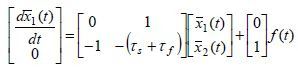


Eliminating ¯*x*_2_(*t*), the approximate slow subsystem and its transfer function are given by

**(18c):**
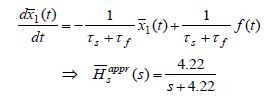


Comparing *H*_*s*_^*appr*^(*s*) and ¯*H*_*s*_^*appr*^(*s*), it appears that ¯*H*_*s*_^*appr*^(*s*) is closer to the exact *H*_*s*_(*s*) from (17) than ¯*H*_*s*_*appr*(*s*).

Note that if one intends to use Simulink, the transfer functions (17) and (18) should be placed in parallel. This parallel structure is convenient for introduction of different saturation elements or time-delay elements along the lines of Horng et al. (9), which in this case can be done independently for pure-slow or pure-fast dynamics. It should be emphasized that introduction of saturation elements leads to nonlinear models, and that the time-delay elements produce in general infinite dimensional models (models described by partial differential equations) and as such they have much more complex dynamics than the model considered in this paper – the dynamics that can display limit cycles (oscillations caused by saturation elements) and even chaotic behavior.

From the slow and fast transfer functions we can get information about how much are the pure slow-slow and pure-fast signals amplified at steady state by finding the corresponding gains. The steady state gains (8), are given by

**(19):**
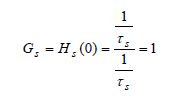


**(20):**
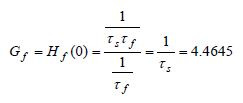


An interesting observation from (20) is that the purefast subsystem steady state gain is reciprocal to the slow time constant. Results in (19) and (20) indicate that pureslow signals will have no amplification at steady state and that pure-fast signals will be considerably (4.4645 times) amplified at steady state.

The original variables *x*_1_(*t*)= *y*(*t*) and *x*_2_(*t*) =*dy*(*t*)/*dt* are obtained from (12) and (13) as follows

**(21):**
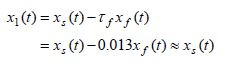


**(22):**
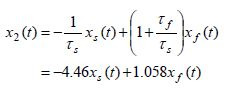


**Figure 1.**
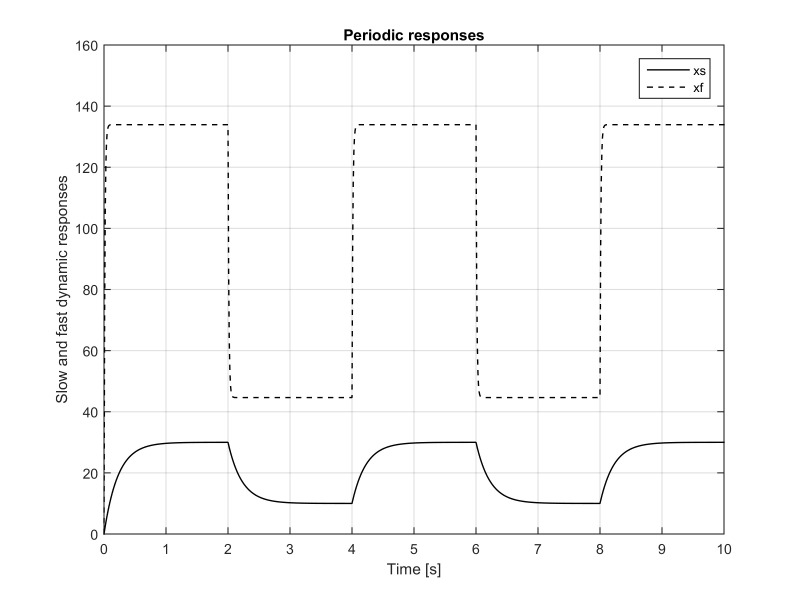
The responses of the pure-slow *x*_*s *_(*t*) and pure-fast *x *_*f *_(*t*) variables in the interval of 10 seconds assuming zero initial conditions. It can be observed from this picture that the eye stimuli in the range of 10° to 30° generate the pure-fast component in the range from 44.6° to133.9°. The figure shows also that the pure-slow component remains in the same range as the input signal, that is, from 10° to 30°.

**Figure 2.**
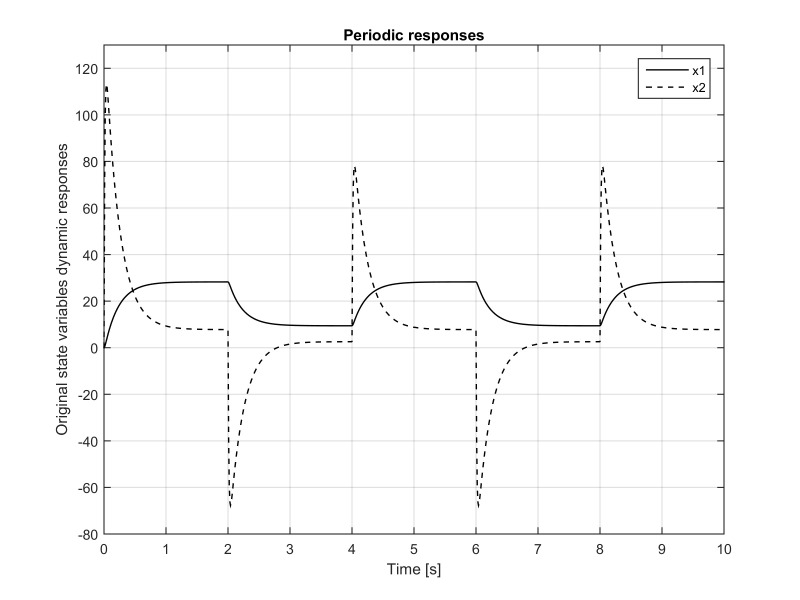
The variables *x*_1_(*t*) and *x*_2_(*t*) as functions of time. It can be observed that the first peak of *x*_2_(*t*) is around 116°/s and that the follow up peaks are around 79°/s . This is caused due to different initial conditions at *t *=0 and *t *=4 . It was shown in the paper that *x*_1_(4) = 9.4 and *x*_2_(4) = 2.6°/s. Due to the input signal decrease from 30° to10°, the fast variable takes a large negative value of ≈-67°/s . During the half period of two seconds *x*_2_(*t*) changes very drastically, from positive 79°/s to negative ≈-67°/s , producing the absolute change of ≈146°/s

**Figure 3.**
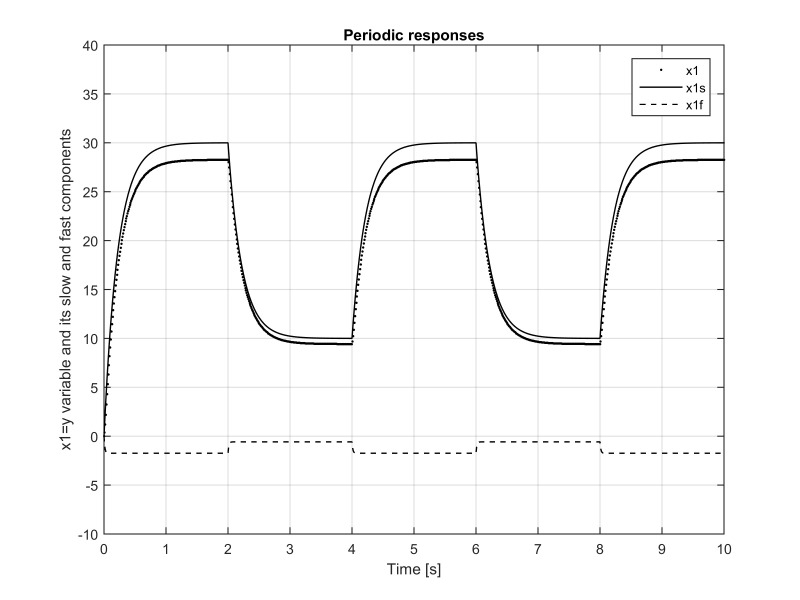
Eye position *x*_1_(*t*) and its pure-slow and pure-fast components. Due to the fact that the slow variable is dominated by its pure-slow components and that it has a negligible contribution of the pure-fast component, the figure shows that practically *x*_1_(*t*) ≈ *x*_1*s *_(*t*), which was also verified analytically in formula (21).

**Figure 4.**
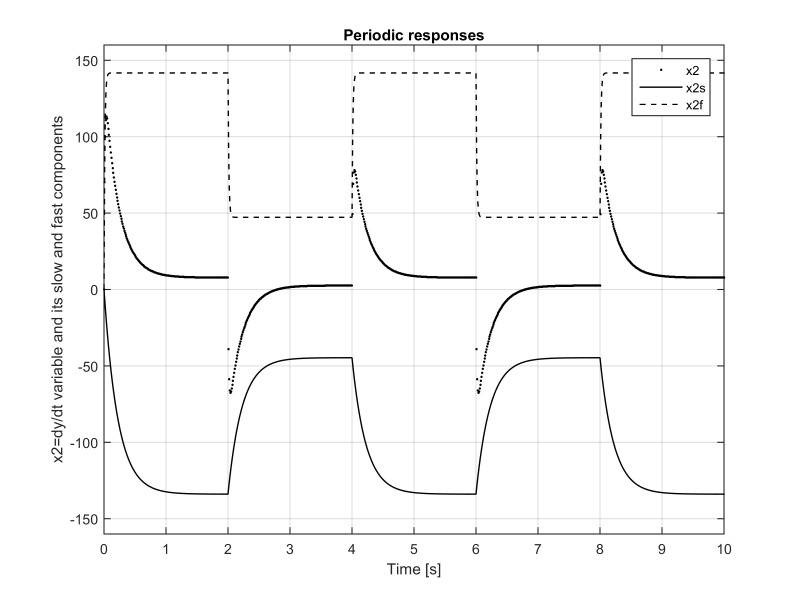
Eye angular velocity *x*_2_(*t*) as a function of time. It can be seen that its pure-fast component *x*_2 *f *_(*t*) very quickly, in several milliseconds, reaches steady state with a very high value of around *x*_2f_^max^=140°/s. When the stimuli changes instantly from 30° to 10°, *x*_2 *f *_(*t*) drops within several milliseconds to a little bit below *x*_2f_^min^=50°/sec. The pure-slow component *x*_2*s *_(*t*) goes in the opposite direction and reaches in less than a second *x*_2_^min^_*s *_=-130°/s. These two components form *x*_2_(*t*)= *x*_2*s*_(*t*)+ *x*_2*f *_(*t*)and together produce at steady state ≈10°/s. Without the pure-slow/pure-fast decomposition, one would not be able to see these violent components of the eye movement dynamics.

The simulation results of (15)-(16) and (21)-(22), assuming zero initial conditions, that is, *x*_1_(0)=0 and *x*_2_(0)=0, are presented in Figs. 1-4.

## Discussion of the obtained simulation results

The dynamic responses of the pure-slow *x*_*s*_(*t*) and pure-fast *x *_*f *_(*t*) variables in the time interval of 10 seconds are presented in Figure 1. It can be observed from this picture that the eye stimuli in the range of 10° to 30°, due to amplification at steady state as given by (20) generate the pure-fast component in the range from 4.4645x10°= 44.645° to 4.4645x30°=133.9353°. The same figure shows that the pure-slow component remains in the same range as the input signal, that is, from 10° to 30°, due to the fact that the pure-slow subsystem steady state gain is*G*_*s *_=1.

The eye position in the original coordinates *x*_1_(*t*), and the eye original coordinates angular velocity (the time rate of the position change) *x*_2_(*t*) are plotted in Figure 2. It should be observed that the *first* peak of *x*_2_(*t*) is around

116°/s and that the follow up peaks are around79°/s. This is caused due to different initial conditions at *t *=0 and *t *= 4,8. We started simulation with zero initial conditions, that is, for the first period the initial conditions are *x*_1_(0)=0° and *x*_2_(0)=0°. For the second period, the initial conditions obtained from formulas (21) and (22) are non-zero and given by

**(23):**
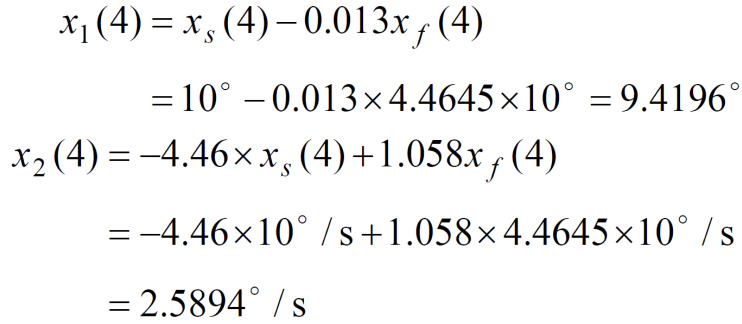


In addition, due to the input signal decrease from 30° to10°, the fast component takes a large negative value of ≈-67°/s. Hence, during the half period of two seconds, the eye angular velocity changes very drastically, from positive 79°/s to negative ≈-67°/s , producing the absolute angular velocity change of ≈146°/s.

The slow variable (eye position) *x*_1_(*t*) and its pureslow and pure-fast components are presented in Figure 3. Due to the fact that the slow variable is dominated by its pure-slow component and that it has a negligible contribution of the pure-fast component, the figure shows that practically *x*_1_(*t*)≈ *x*_1*s*_(*t*), could have been also verified analytically using formula (21).

Much more interesting situation is with the fast variable *x*_2_(*t*) that represents the eye angular velocity, see Figure 4. It can be seen from this figure that its pure-fast component *x*_2 *f *_(*t*) very quickly, in several milliseconds, reaches steady state with a maximum value of around *x*_2*f *_^max^=140°/s. When the stimuli changes instantly from 30° to 10° , the variable *x*_2 *f *_(*t*) drops within several milliseconds to a little bit below *x*_2*f *_^min^= 50°/sec. On the other hand, the pure-slow component *x*_2*s*_(*t*) goes in the opposite direction and reaches in less than a second *x*_2*s*_^min^=-130°/s. These two components form *x*_2_(*t*)= *x*_2*s *_(*t*)+ *x*_2 *f *_(*t*) and together produce at steady state ≈10°/s for the eye angular velocity. Without the pure-slow/pure-fast decomposition, one would not be able to see these violent component in the disparity convergence of the eye movement dynamics.

## Conclusions

It was shown that the fast component of the eye dynamics displays very fast and abrupt changes due to considered time varying stimuli as demonstrated in Figures 2 and 4. The angular velocity, due to the change of the stimuli force of 20 degrees (from 30° to10°), displays large variations of more than 140°/s, as shown in Figure 4. This large change could have been restricted by introduction of a saturation element. However, that will lead to a new nonlinear mathematical model different than the linear second-order mathematical model considered in this paper. Such nonlinear models are not the subject of this paper, and they will be interesting for future research.

## Ethics and Conflict of Interest

The author(s) declare(s) that the contents of the article are in agreement with the ethics described in http://biblio.unibe.ch/portale/elibrary/BOP/jemr/ethics.html and that there is no conflict of interest regarding the publication of this paper.
